# Associations of TNF-α, MIF, and cortisol with cognitive function in patients with bipolar disorder during acute manic episodes: a short-term follow-up study

**DOI:** 10.3389/fpsyt.2026.1863310

**Published:** 2026-06-29

**Authors:** Heng Tang, Siwei Lin, Xiaoxuan Liu, Jin Chen, Xiaowei Zuo

**Affiliations:** 1 Department of Psychiatry, Xuzhou Medical University, Xuzhou, Jiangsu, China; 2 Department of Psychiatry, The Affiliated Xuzhou Oriental Hospital of Xuzhou Medical University, Xuzhou, Jiangsu, China

**Keywords:** bipolar disorder, cognitive function, cortisol, macrophage migration inhibitory factor, tumor necrosis factor-α

## Abstract

**Background:**

Bipolar disorder (BD) is frequently accompanied by cognitive impairment, and growing evidence suggests that immune-inflammatory activation and hypothalamic-pituitary-adrenal axis dysregulation may contribute to its pathophysiology. This study aimed to examine the associations of tumor necrosis factor-α (TNF-α), macrophage migration inhibitory factor (MIF), and cortisol (COR) with cognitive function in patients with BD during manic episodes and to characterize their short-term changes.

**Methods:**

In this short-term follow-up study, 53 patients with BD during manic episodes and 53 healthy controls (HCs) were enrolled. Plasma TNF-α, MIF, and COR levels were measured using enzyme-linked immunosorbent assay. Cognitive function was assessed using the Chinese Brief Cognitive Test, including information processing speed (IPS), executive function (EF), sustained attention (SAT), and working memory (WM). Patients were evaluated at baseline and after 8 weeks of treatment, whereas HCs were assessed once at baseline. Group comparisons and biomarker–cognition correlation analyses were performed. Multiple testing in the correlation analyses was controlled using the Benjamini–Hochberg false discovery rate (FDR) procedure.

**Results:**

At both baseline and follow-up, patients with BD had significantly lower IPS, EF, SAT, and WM scores, but significantly higher plasma TNF-α, MIF, and COR levels, than HCs. After 8 weeks of treatment, cognitive scores in the BD group improved significantly, whereas reductions in TNF-α, MIF, and COR did not reach statistical significance. In exploratory unadjusted Pearson analyses, several biomarker–cognition associations survived FDR correction. However, in the primary adjusted partial correlation analyses, only the negative association between TNF-α and WM remained significant after adjustment for covariates and FDR correction at both baseline and follow-up.

**Conclusion:**

Patients with BD during manic episodes exhibited widespread cognitive impairment accompanied by elevated inflammatory and neuroendocrine markers. TNF-α showed the most robust association with working memory after adjustment for covariates and correction for multiple comparisons. Associations involving MIF or cortisol and executive function should be interpreted as exploratory and require validation in larger longitudinal studies.

## Introduction

Bipolar disorder (BD) is a chronic and severe mental disorder primarily characterized by recurrent episodes of mania or hypomania alternating with depressive episodes. It typically begins in adolescence or early adulthood and is associated with high rates of relapse, disability, and mortality, although clinical diagnosis and standardized treatment remain insufficient in many settings (1). Epidemiological studies indicate that BD affects approximately 0.5%–1% of the global population (2). Beyond recurrent mood symptoms, BD is frequently accompanied by cognitive impairment, which imposes a substantial medical and socioeconomic burden on patients, their families, and society (3). Current evidence suggests that abnormalities in stress regulation, immune-inflammatory activity, and related neurobiological processes may contribute to the pathogenesis and progression of BD (4, 5). Cognitive impairment is increasingly recognized as an important clinical dimension of BD rather than merely a secondary manifestation of mood symptoms. Previous studies have shown that patients with BD commonly exhibit impairments across multiple cognitive domains, including executive function, attention, and memory, and that these deficits may persist during both acute episodes and periods of euthymia, at least to some extent independent of current mood symptoms (6–8).

Importantly, cognitive dysfunction in BD appears to vary across mood states rather than remaining entirely stable throughout the course of illness. Although cognitive deficits are also detectable during remission, several studies have shown that acute manic or hypomanic episodes are associated with relatively more prominent impairments, especially in verbal memory, working memory, attention, and executive function. Therefore, focusing on patients during manic episodes may help capture a clinically meaningful stage in which cognition-related biological abnormalities are more readily detectable (9–11). These deficits may further affect daily living, occupational functioning, and social adaptation, thereby becoming important determinants of psychosocial recovery and long-term prognosis (12–14).

The biological mechanisms underlying cognitive impairment in BD are complex and likely involve the interaction of genetic susceptibility, neuroimmune-inflammatory abnormalities, neuroendocrine imbalance, and neural circuit dysfunction (15). Among these factors, tumor necrosis factor-α (TNF-α), a classical pro-inflammatory cytokine, has been reported to be abnormally elevated in patients with BD and to be consistently associated with cognitive impairment (16, 17). Previous studies have further suggested that TNF-α levels are significantly increased in patients with BD, particularly during manic episodes, and are associated with cognitive deficits (18). In addition to TNF-α, macrophage migration inhibitory factor (MIF), a multifunctional pro-inflammatory cytokine with broad immunoregulatory properties, has attracted increasing attention. MIF may promote neuroinflammatory responses through chemokine-like activity and may contribute to neurotoxicity and apoptosis through its enzymatic properties, suggesting a potential role in cognitive impairment-related pathways (19). Although evidence from other severe mental disorders indicates that elevated MIF levels may be associated with cognitive impairment and cognitive changes after treatment (20), direct evidence regarding the relationship between MIF and cognitive function in BD remains limited.

In parallel with immune-inflammatory abnormalities, neuroendocrine dysregulation may represent another closely related pathway linking BD to cognitive impairment (21). The hypothalamic-pituitary-adrenal (HPA) axis is the central neuroendocrine system that regulates the body’s stress response, and cortisol (COR) serves as a key biological marker of HPA axis function (22). Cortisol receptors are widely distributed in brain regions involved in cognitive processing, such as the hippocampus and prefrontal cortex (23). Persistent or dysregulated cortisol exposure may affect neuronal function, dendritic remodeling, and brain structure, thereby contributing to impairment in cognitive domains such as memory and executive function (24, 25). Importantly, immune-inflammatory and HPA-axis abnormalities are closely interconnected. Pro-inflammatory cytokines can influence HPA-axis activity, whereas impaired glucocorticoid-mediated negative feedback may further amplify inflammatory signaling (26). This bidirectional interaction provides a plausible framework for understanding how inflammatory and neuroendocrine abnormalities may jointly contribute to cognitive dysfunction in BD.

Although previous studies have provided valuable evidence linking inflammatory or neuroendocrine markers to cognitive impairment in BD, much of the existing literature remains cross-sectional and heterogeneous across biomarkers, mood states, and cognitive domains (17, 27–29). Consequently, short-term longitudinal data are still needed to clarify whether cognitive changes during treatment occur in parallel with, or diverge from, changes in peripheral inflammatory and HPA-axis-related markers. Building on this existing literature, the present short-term follow-up study examined plasma TNF-α, MIF, and COR levels and cognitive performance in patients with BD during acute manic episodes before and after 8 weeks of treatment. This study aimed to characterize short-term changes in these biomarkers and cognitive domains and to explore whether specific biomarker–cognition associations remain evident after adjustment for relevant covariates and correction for multiple comparisons.

## Materials and methods

### Participant recruitment

The patient cohort in this study was recruited from individuals with manic episodes of bipolar disorder who were treated at the Affiliated Xuzhou Oriental Hospital of Xuzhou Medical University. The patient group ultimately enrolled 53 participants. The inclusion criteria were as follows: (1) fulfillment of the ICD-10 diagnostic criteria for a manic episode of BD; (2) a Bech-Rafaelsen Mania Scale (BRMS) score ≥ 10; (3) age between 18 and 50 years, right-handedness, and Han ethnicity; (4) an educational level of junior high school or above; and (5) provision of written informed consent and voluntary participation in the study. The exclusion criteria were as follows: (1) comorbid psychiatric disorders meeting ICD-10 diagnostic criteria; (2) receipt of nonconvulsive electroconvulsive therapy within the previous month; (3) the presence of severe physical illness or active infectious disease (e.g., pneumonia or cellulitis); (4) intellectual disability; (5) use of corticosteroids or other medications that may affect cytokine levels within the previous 2 months, such as nonsteroidal anti-inflammatory drugs (NSAIDs) or antibiotics; (6) pregnancy or lactation; and (7) a history of severe or persistent substance abuse.

The healthy control (HC) group consisted of 53 individuals who underwent health examinations at the Health Screening Center of the same hospital during the same period. The inclusion criteria were as follows: (1) age between 18 and 50 years, right-handedness, Han ethnicity, and either sex; (2) no history of BD or other psychiatric disorders; (3) an educational level of junior high school or above; and (4) provision of written informed consent and voluntary participation in the study. The exclusion criteria for the healthy control group were identical to those for the patient group.

This study was approved by the Medical Ethics Committee of the Affiliated Xuzhou Oriental Hospital of Xuzhou Medical University (Approval No. 2025011004). Written informed consent was obtained from all participants after they had received a full explanation of the study.

### Clinical assessment and general data collection

General demographic data were collected from both groups, including age, sex, and years of education. The patient group underwent a baseline assessment before treatment and a follow-up assessment 8 weeks after treatment, whereas the healthy control group underwent a single baseline assessment. The severity of manic symptoms in the patient group was assessed using the BRMS. The BRMS is a clinician-rated scale developed to evaluate manic symptom severity. It consists of 11 items, each scored from 0 to 4, with total scores ranging from 0 to 44; higher scores indicate more severe manic symptoms. Previous studies have supported its inter-observer reliability and clinical utility in assessing manic states (30). In this study, a BRMS score ≥10 was used as one of the inclusion criteria for manic episodes. Clinical response at follow-up was operationally defined as a reduction of more than 50% in BRMS score from baseline to week 8. Because the BRMS primarily assesses manic symptoms, full euthymia was not defined solely on the basis of BRMS scores. For descriptive purposes, BRMS <7 was used to indicate remission-level manic symptoms at follow-up. All clinical data were collected by researchers who had received standardized training and followed a predetermined protocol to ensure consistency and completeness of data collection.

### Cognitive assessment

The Chinese Brief Cognitive Test (CBCT) was used to assess participants’ cognitive function. The CBCT consists of four subtests: Connecting Test A, Symbol Coding, Sustained Performance, and Digit Span, each of which is designed to evaluate a distinct cognitive domain. Specifically, Connecting Test A primarily assesses information processing speed, visual scanning ability, and cognitive flexibility; Symbol Coding primarily assesses attention, information processing speed, and executive functions related to task switching; Sustained Performance primarily assesses sustained and selective attention; and Digit Span primarily assesses auditory verbal memory and working memory. The four domain scores generated by the CBCT correspond to information processing speed (IPS), executive function (EF), sustained attention (SAT), and working memory (WM), respectively. Higher domain scores indicate better cognitive performance. Because the present study focused on domain-specific cognitive performance, the four domain scores were analyzed rather than a total CBCT score. The CBCT has demonstrated good reliability and validity (31). The patient group completed cognitive assessments at baseline and again after 8 weeks of treatment, whereas the healthy control group completed a single cognitive assessment at baseline.

### Measurement of plasma biomarkers

In the patient group, 5 mL of fasting venous blood was collected in the morning at baseline and again after 8 weeks of standardized clinical treatment; in the healthy control group, blood samples were collected once at baseline. After collection, the samples were centrifuged at 3,500 rpm for 10 min, and the plasma was separated and stored at −80 °C for subsequent analysis. Plasma levels of MIF, TNF-α, and COR were measured using enzyme-linked immunosorbent assay (ELISA). The ELISA kits and related reagents were purchased from Shanghai Fenji Biotechnology Co., Ltd. All assays were performed in strict accordance with the manufacturer’s instructions.

### Sample size estimation

Sample size estimation was performed using G*Power 3.1 (32). Because the healthy control group underwent only a baseline assessment, the calculation was based on the primary within-group longitudinal analysis in the BD group. Repeated-measures ANOVA (within factors) was selected, with the following parameters: effect size f = 0.25, α = 0.05, power = 0.80, two repeated measurements, correlation among repeated measures = 0.50, and ϵ = 1. The estimated minimum sample size was 34 participants. A total of 53 patients with BD were ultimately included, exceeding the estimated minimum. This sample size estimation was based on the primary within-group longitudinal analysis of cognitive changes and was not specifically designed to ensure adequate statistical power for the biomarker–cognition correlation analyses. Therefore, the correlation and partial correlation analyses should be regarded as exploratory.

### Statistical analysis

Statistical analyses were performed using SPSS version 26.0. The normality of continuous variables was assessed using the Shapiro–Wilk test. Continuous variables with an approximately normal distribution were expressed as mean ± standard deviation ( $\bar{x}$ ± s), whereas non-normally distributed variables were presented as median (interquartile range). Between-group comparisons were performed using the independent-samples t-test for normally distributed variables and the Mann–Whitney U test for non-normally distributed variables. Within-group comparisons before and after treatment in the BD group were conducted using the paired-samples t-test. Categorical variables were expressed as frequencies (%) and compared using the chi-square (*χ²*) test. Pearson correlation analysis was used as an exploratory unadjusted analysis to examine the crude associations between plasma biomarkers and cognitive performance, and selected associations were visualized using scatter plots with fitted regression lines. Partial correlation analysis was considered the primary inferential analysis and was performed after adjustment for sex, age, years of education, BMI, and illness duration. Because illness duration was not normally distributed, sensitivity analyses were additionally performed using log-transformed illness duration in the adjusted partial correlation models.

To account for multiple testing in the biomarker–cognition correlation analyses, p values were adjusted using the Benjamini–Hochberg false discovery rate (FDR) procedure. FDR correction was applied separately to the exploratory Pearson correlation analyses and to the primary adjusted partial correlation analyses. Each set included 24 biomarker–cognition tests derived from 2 time points, 4 cognitive domains, and 3 biomarkers. FDR-adjusted *q* values < 0.05 were considered statistically significant. All statistical tests were two-tailed.

## Results

### General information, clinical characteristics, and pharmacotherapy

A comparison of the general demographic and clinical characteristics of the two groups, as well as the pharmacotherapy regimen in the patient group, is presented in **Table 1**. Chi-square analysis showed no significant difference in sex distribution between the two groups (*p* > 0.05). Independent-samples t-tests showed no significant differences in age or body mass index (BMI) between the two groups (*p* > 0.05). However, the Mann-Whitney U test showed that the healthy control (HC) group had significantly more years of education than the BD group (*p* < 0.05).

**Table 1:** Demographic and clinical characteristics of the BD and HC groups, and pharmacotherapy in the BD group.

Variable	BD (n = 53)	HC (n = 53)	t/Z/*χ²*	*p*
Demographic characteristics				
Sex (male/female), n	30/23	25/28	0.945	0.331
Age	32.87 ± 11.51	32.53 ± 6.83	0.185	0.854
Years of education	12.00(9.00,15.00)	16.00(16.00,17.00)	-8.111	<0.001
BMI	24.67 ± 4.48	24.22 ± 2.73	0.631	0.530
Clinical characteristics				
Illness duration	8.00(2.50,13.50)	-	-	-
Baseline BRMS	23.87 ± 5.79	-	-	-
Follow-up BRMS	11.04 ± 3.83	-	-	-
Treatment-related variables				
Lithium treatment, n (%)				
Lithium treatment	45(85%)	-	-	-
Non-lithium treatment	8(15%)	-	-	-
Chlorpromazine-equivalent dose	366.75 ± 146.98	-	-	-

BD, bipolar disorder; HC, healthy controls; BMI, body mass index; BRMS, Bech-Rafaelsen Mania Scale.

Continuous variables are presented as mean ± SD or median (IQR), as appropriate, and categorical variables as n (%). Between-group comparisons were performed using the independent-samples t-test for normally distributed variables, the Mann–Whitney U test for non-normally distributed variables, and the chi-square (*χ²*) test for categorical variables. The statistic column reports *t*, *Z*, or *χ²* values as appropriate. *p* < 0.05 was considered statistically significant.

### Pharmacotherapy and correlations with follow-up biomarkers and cognitive performance

In this study, all patients with BD received combination therapy that included second-generation antipsychotics (SGAs), and 45 patients (84.9%) additionally received lithium carbonate as a mood stabilizer.

To assess the potential impact of antipsychotic dosage on the study findings, the doses of the relevant antipsychotic agents were standardized to chlorpromazine (CPZ) equivalents, and their correlations with biomarker levels and cognitive performance were further analyzed. Pearson correlation analysis showed that CPZ-equivalent doses were not significantly correlated with follow-up levels of MIF, TNF-α, or COR, nor with any cognitive measures (all *p* > 0.05; **Supplementary Table 1**).

### Between-group comparisons of plasma TNF-α, MIF, COR, and cognitive performance

**Figure 1:**
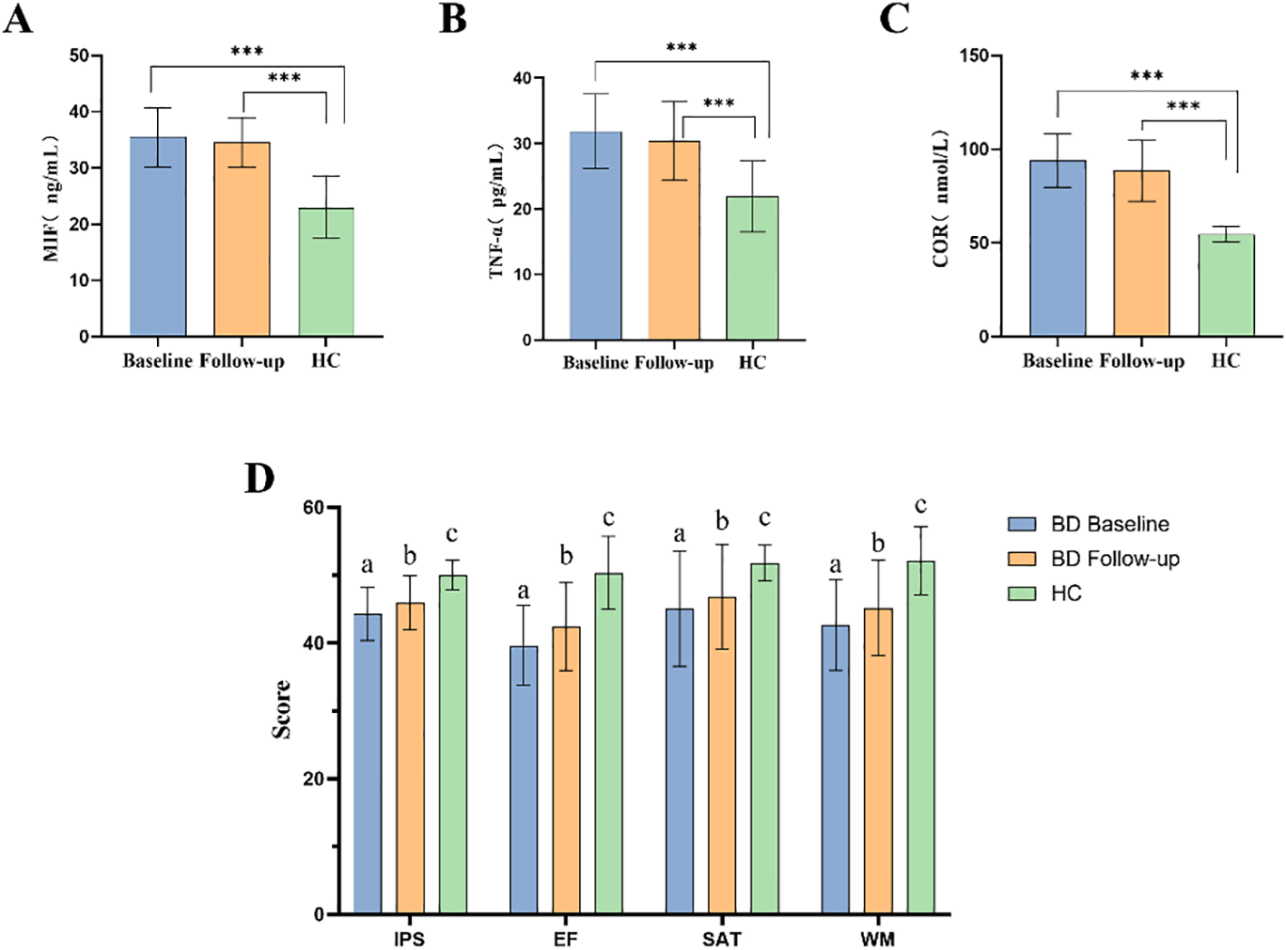
Comparisons of plasma MIF, TNF-α, COR, and cognitive performance across the BD baseline, BD follow-up, and HC groups. **(A)** MIF; **(B)** TNF-α; **(C)** COR; **(D)** cognitive performance. Data are presented as mean ± SD. *p < 0.05; ***p < 0.001; ns, not significant. Different lowercase letters indicate statistically significant differences among groups within each cognitive domain (*p* < 0.05).

**Figure 1** and **Supplementary Table 2** present the between-group comparisons of plasma biomarkers and cognitive performance. Independent-samples t-tests showed that, at both baseline and follow-up, the HC group had significantly higher IPS, EF, SAT, and WM scores, but significantly lower plasma MIF, TNF-α, and COR levels, than the BD group (all *p* < 0.05).

To account for the significant between-group difference in years of education, ANCOVA was additionally performed for baseline cognitive measures, with group and sex as fixed factors and age, body mass index, and years of education as covariates. The adjusted group effect remained significant across all cognitive domains (all *p* < 0.05; **Supplementary Table 3**), suggesting that the between-group differences in cognitive performance were not explained solely by demographic differences.

### Within-group comparisons of plasma TNF-α, MIF, COR, and cognitive performance in the BD group

**Table 2** presents a comparison of TNF-α, MIF, and COR levels, as well as cognitive measures, in the BD group before and after treatment. Paired-samples t-tests showed that post-treatment IPS, EF, SAT, and WM scores were significantly higher than the corresponding pretreatment scores (all *p* < 0.05). Post-treatment levels of TNF-α, MIF, and COR were lower than pretreatment levels; however, these differences did not reach statistical significance (all *p* > 0.05).

**Table 2:** Longitudinal changes in cognitive performance and plasma biomarker levels in the BD group.

Variable	Baseline	Follow-up	*t*	*p*
Cognition				
IPS	44.3 ± 3.95	45.94 ± 4.01	-5.916	<0.001
EF	39.64 ± 5.88	42.42 ± 6.48	-3.743	<0.001
SAT	45.04 ± 8.50	46.83 ± 7.73	-2.940	0.005
WM	42.66 ± 6.70	45.15 ± 7.01	-3.526	0.001
Biomarkers				
MIF	35.46 ± 5.26	34.51 ± 4.42	1.077	0.286
TNF-α	31.86 ± 5.66	30.38 ± 6.00	1.472	0.147
COR	93.86 ± 14.24	88.39 ± 16.23	1.989	0.052

IPS, information processing speed; EF, executive function; SAT, sustained attention; WM, working memory; MIF, macrophage migration inhibitory factor; TNF-α, tumor necrosis factor-α; COR, cortisol.

Data are presented as mean ± SD. Higher cognitive scores indicate better performance in the corresponding domain. *p* < 0.05 was considered statistically significant.

At the 8-week follow-up, 7 patients (13.2%) had BRMS scores <7, whereas 46 patients (86.8%) still had BRMS scores ≥7. Thus, the 8-week follow-up should be interpreted as a post-treatment clinical response assessment rather than as a euthymic-state assessment. To explore whether the observed cognitive improvement was associated with symptomatic improvement, Spearman correlation analysis was performed between changes in BRMS scores and changes in cognitive measures in the BD group. No significant correlations were found between changes in BRMS scores and changes in IPS (*ρ* = 0.064, *p* = 0.648), EF (*ρ* = 0.193, *p* = 0.167), SAT (*ρ* = 0.125, *p* = 0.373), or WM (*ρ* = -0.023, *p* = 0.870).

### Primary adjusted partial correlation analysis of cognitive performance and plasma TNF-α, MIF, and COR levels

The adjusted partial correlations between cognitive performance and TNF-α, MIF, and COR are presented in **Table 3**. After adjustment for sex, age, years of education, BMI, and illness duration, and after FDR correction for 24 adjusted biomarker–cognition partial correlations, only the negative association between TNF-α and WM remained statistically significant. Specifically, pretreatment TNF-α was negatively correlated with WM scores (*r* = −0.482, *p* = 0.001, FDR-adjusted *q* = 0.012), and this association remained significant at follow-up (*r* = −0.541, *p* < 0.001, FDR-adjusted *q* = 0.012). The associations of COR with EF at baseline (*r* = −0.318, *p* = 0.029, FDR-adjusted *q* = 0.139), MIF with EF at follow-up (*r* = −0.356, *p* = 0.014, FDR-adjusted *q* = 0.084), and COR with EF at follow-up (*r* = −0.363, *p* = 0.012, FDR-adjusted *q* = 0.084) were nominally significant before FDR correction but did not survive correction for multiple comparisons. Sensitivity analyses using log-transformed illness duration as a covariate yielded materially similar results.

**Table 3:** Adjusted partial correlations of cognitive performance with plasma biomarkers in the BD Group After FDR correction.

Time point	Cognitive domain	MIF			TNF-α			COR		
		*r*	*p*	*q*	*r*	*p*	*q*	*r*	*p*	*q*
Baseline	IPS	-0.015	0.923	0.923	0.073	0.624	0.794	0.035	0.814	0.849
	EF	-0.058	0.697	0.794	0.094	0.528	0.794	-.318	0.029	0.139
	SAT	0.087	0.563	0.794	0.272	0.064	0.187	-0.078	0.602	0.794
	WM	-0.070	0.642	0.794	-.482	0.001	0.012	-0.267	0.070	0.187
Follow-up	IPS	-0.112	0.455	0.794	0.052	0.728	0.794	0.184	0.215	0.491
	EF	-.356	0.014	0.084	-.280	0.056	0.187	-.363	0.012	0.084
	SAT	-0.098	0.511	0.794	0.065	0.665	0.794	0.092	0.541	0.794
	WM	-0.280	0.057	0.187	-.541	<0.001	0.012	-0.181	0.225	0.491

IPS, information processing speed; EF, executive function; SAT, sustained attention; WM, working memory; MIF, macrophage migration inhibitory factor; TNF-α, tumor necrosis factor-α; COR, cortisol.

Partial correlations were adjusted for sex, age, years of education, body mass index, and illness duration. The Benjamini–Hochberg false discovery rate (FDR) procedure was applied to 24 adjusted biomarker–cognition partial correlations. FDR-adjusted q < 0.05 was considered statistically significant. Statistically significant correlations after FDR correction are shown in bold.

### Exploratory unadjusted pearson correlations of cognitive performance with plasma TNF-α, MIF, and COR

**Figure 2:**
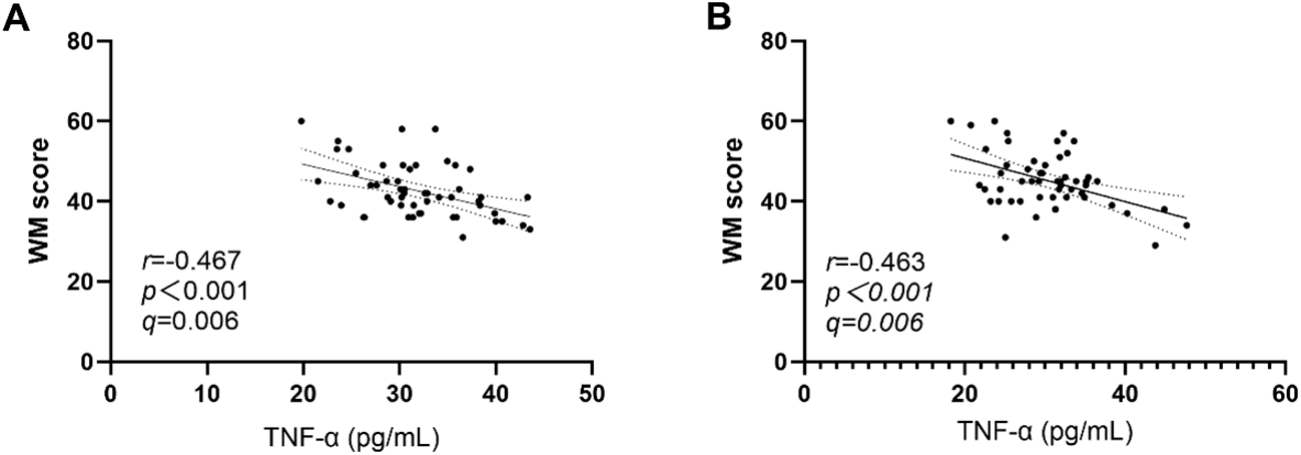
Exploratory unadjusted Pearson correlations between plasma TNF-α and working memory in the BD group. **(A)** Baseline TNF-α and working memory; **(B)** follow-up TNF-α and working memory. Pearson correlation coefficients are shown in each panel. The solid lines represent fitted linear regression lines, and the shaded areas indicate the corresponding 95% confidence intervals. FDR-adjusted *q* values were calculated across 24 exploratory Pearson biomarker–cognition correlations. In the primary adjusted partial correlation analysis, the association between TNF-α and working memory also remained significant at baseline (*r* = −0.482, *p* = 0.001, FDR-adjusted *q* = 0.012) and follow-up (*r* = −0.541, *p* < 0.001, FDR-adjusted *q* = 0.012).

The exploratory unadjusted Pearson correlations of TNF-α, MIF, and COR with cognitive performance before and after treatment in the BD group are summarized in **Supplementary Table 4**. The associations between TNF-α and WM at baseline and follow-up are visualized in **Figure 2**. After FDR correction for 24 exploratory Pearson biomarker–cognition correlations, pretreatment TNF-α levels remained negatively correlated with WM scores (*r* = −0.467, *p* < 0.001, FDR-adjusted *q* = 0.006), and pretreatment COR levels remained negatively correlated with EF scores (*r* = −0.411, *p* = 0.002, FDR-adjusted *q* = 0.010). At follow-up, EF scores remained negatively correlated with MIF (*r* = −0.429, *p* = 0.001, FDR-adjusted *q* = 0.006) and COR (*r* = −0.441, *p* < 0.001, FDR-adjusted *q* = 0.006), and TNF-α levels remained negatively correlated with WM scores (*r* = −0.463, *p* < 0.001, FDR-adjusted *q* = 0.006). The unadjusted association between TNF-α and EF at follow-up was nominally significant before correction (*r* = −0.321, *p* = 0.019) but did not survive FDR correction (FDR-adjusted *q* = 0.076).

## Discussion

This longitudinal study investigated the relationships among peripheral inflammatory factors, cortisol levels, and cognitive function in patients with BD during manic episodes. Compared with healthy controls, patients with BD showed significant impairments in information processing speed, executive function, sustained attention, and working memory at both baseline and follow-up. These findings indicate that cognitive dysfunction in this population is widespread and remains evident after short-term treatment, consistent with previous studies (33). Plasma TNF-α, MIF, and COR levels were also significantly higher in the BD group than in healthy controls at both time points, indicating persistent immune-inflammatory activation and HPA axis dysfunction during the manic phase of BD (34). After 8 weeks of treatment, cognitive performance improved significantly, whereas reductions in TNF-α, MIF, and COR did not reach statistical significance. In the biomarker–cognition analyses, the most robust finding was the negative association between TNF-α and working memory, which remained significant after adjustment for covariates and FDR correction at both baseline and follow-up. By contrast, associations involving MIF or COR and executive function were nominally significant but did not survive FDR correction. These findings suggest that cognitive impairment in BD during manic episodes may persist beyond short-term symptomatic improvement and may be partly related to immune-inflammatory and neuroendocrine abnormalities. Among the examined biomarkers, TNF-α showed the most robust association with working memory, whereas findings involving MIF or COR should be considered exploratory.

The present findings indicate that, at baseline, patients with BD during manic episodes performed significantly worse than healthy controls across multiple cognitive domains, including information processing speed, executive function, sustained attention, and working memory. These differences remained evident at the 8-week follow-up, further supporting the presence of widespread cognitive impairment in this population. This result is consistent with the overall conclusions of recent studies (35, 36). A 10-year follow-up study by Flaaten et al. showed that patients with BD may exhibit long-term stability or even mild improvement in certain cognitive domains. Although their overall cognitive performance remained lower than that of healthy controls, their cognitive trajectories generally paralleled those of the healthy population, with no clear evidence supporting a universally progressive decline in cognitive function (37). Taken together, these findings suggest that cognitive impairment is not merely a transient manifestation during acute episodes, but is more likely a core component of the clinical phenotype of BD. This interpretation is also consistent with evidence suggesting that cognitive dysfunction in BD has substantial implications for psychosocial functioning, daily living, and long-term recovery. Cognitive performance has been linked not only to symptom burden but also to functional outcome, and recent reviews have emphasized that cognitive heterogeneity itself may be clinically meaningful in BD (7, 13, 38). Miskowiak et al. conducted cognitive screening in patients with BD who had received optimized treatment in a specialized outpatient clinic and found that, although most patients had achieved complete or partial remission, a substantial proportion still exhibited global or selective cognitive impairment. Furthermore, the screening procedure demonstrated good clinical feasibility and relevance (39). Therefore, the widespread cognitive impairment observed in the present study should not be regarded merely as a concomitant feature of the acute phase of illness, but rather as a key clinical dimension influencing symptom remission, functional recovery, and long-term prognosis.

In addition to persistent cognitive impairment, the present study also demonstrated sustained elevations of plasma TNF-α, MIF, and COR in patients with BD relative to healthy controls at both baseline and the 8-week follow-up, suggesting that peripheral immune-inflammatory activation and neuroendocrine dysfunction may not fully resolve even after short-term treatment. Common immunological alterations in affective disorders include elevated levels of pro-inflammatory cytokines, particularly abnormalities in markers such as TNF-α and IL-6. Inflammatory responses may contribute to the pathophysiology of the disorder by affecting neurotransmitter transmission, microglial activation, brain plasticity, and HPA axis regulation (40). TNF-α and its soluble receptor are generally elevated in patients with BD, supporting the involvement of TNF-related inflammatory pathways in the pathophysiology of BD (16). In the present study, TNF-α levels remained consistently higher than those in healthy controls, suggesting sustained peripheral immune activation during manic episodes. This finding is broadly consistent with previous clinical studies reporting enhanced inflammatory responses during manic or hypomanic phases (16, 41). In addition to TNF-α, MIF levels were also higher in the patient group than in healthy controls at both baseline and follow-up. Compared with TNF-α, direct clinical studies on MIF in BD remain relatively limited. However, some studies have reported that the MIF-173G/C variant is associated with the onset of BD and certain clinical features, suggesting that MIF-related pathways may contribute to susceptibility to BD and clinical heterogeneity (42). In the present study, persistently elevated MIF levels are more likely to reflect a sustained inflammatory amplification state in patients with BD during manic episodes, although its direct role in BD-related cognitive impairment still requires further validation in longitudinal clinical studies.

While TNF-α and MIF reflect immune-inflammatory activation, COR provides complementary information regarding HPA-axis function. In the present study, cortisol levels were higher in patients with BD than in healthy controls at both baseline and follow-up, suggesting that HPA axis abnormalities may persist throughout the short-term recovery period after a manic episode rather than being confined to the acute phase. A longitudinal study of hair cortisol found that patients with BD exhibited significantly greater fluctuations in cortisol over a one-year period than healthy controls, suggesting a slowly evolving and dynamic imbalance of the HPA axis in BD (22). Persistent or dysregulated cortisol exposure has been associated with impaired emotional stability and cognitive decline (43). More importantly, the inflammatory system and the HPA axis do not operate independently. Persistent inflammation can weaken glucocorticoid-mediated negative feedback, whereas prolonged exposure to high glucocorticoid levels, together with cytokine-mediated impairment of glucocorticoid receptor function, may further amplify pro-inflammatory signaling, thereby creating a pathological cycle in which immune-inflammatory and neuroendocrine abnormalities mutually reinforce one another (44). Therefore, the persistently elevated TNF-α, MIF, and COR levels observed during follow-up in this study, relative to those in healthy controls, may reflect not merely a transient biological response during the acute manic phase, but rather a more enduring imbalance in immune-inflammatory and neuroendocrine homeostasis in patients with BD. Taken together with previous studies, this imbalance may provide a biological context for understanding cognitive impairment in BD, although direct mechanistic evidence remains to be established.

After correction for multiple comparisons, the evidence for biomarker–cognition associations was strongest for TNF-α and working memory rather than for a broader pattern of domain-specific biomarker effects. Specifically, TNF-α showed a relatively stable negative association with working memory both before and after treatment, and this relationship remained significant after adjustment for sex, age, years of education, BMI, illness duration, and FDR correction, suggesting that TNF-α may be the most stable candidate marker associated with cognitive impairment in the present study. Coelho et al. reported a relatively consistent association between inflammation and cognitive impairment in BD, with TNF-α, CRP, IL-6, and IL-1RA being the markers most frequently associated with executive dysfunction, reduced processing speed, and memory impairment. However, because most existing studies are cross-sectional in design, the specific links between different inflammatory markers and distinct cognitive domains still require further validation in longitudinal studies (17). Previous studies have shown that elevated peripheral inflammation is associated with impaired executive function and working memory in patients with BD, as well as with abnormal corticostriatal functional connectivity and white matter microstructural alterations (45–47). In particular, inflammatory abnormalities may be related to dysfunction within the prefrontal-limbic and cognitive control networks. Because working memory depends heavily on the integrative function of prefrontal-related networks, the consistent negative association between TNF-α and working memory observed in this study may reflect the persistent impact of inflammatory burden on prefrontal cognitive control circuitry (48, 49). More broadly, accumulating evidence supports a relationship between inflammatory burden and cognitive dysfunction in BD, although the magnitude and domain-specificity of these associations remain variable across studies. Recent work has suggested that inflammation-related cognitive abnormalities in BD may involve executive functioning, processing speed, and memory, but that not all biomarkers show the same degree of consistency across clinical states (29, 50).

The associations of COR and MIF with executive function should be interpreted more cautiously. Although COR with EF at baseline and MIF/COR with EF at follow-up were nominally significant in the adjusted analyses, these associations did not survive FDR correction in the primary partial correlation models. Previous studies have shown that patients with BD exhibit greater long-term fluctuations in cortisol levels, and that elevated or dysregulated cortisol may be associated with impairment in cognitive functions such as memory (25). Therefore, the observed COR–EF pattern may be biologically plausible in the context of chronic stress burden and prefrontal vulnerability, but it should be considered exploratory rather than confirmatory. Similarly, MIF showed a nominal negative association with executive function during follow-up; however, based on the current literature, direct clinical evidence linking MIF to cognitive impairment in BD remains limited. Accordingly, the present finding should be viewed as preliminary. At present, MIF-related evidence in BD is still largely indirect, and its cognitive relevance is more strongly supported by broader mechanistic reviews and by adjacent evidence from other severe mental disorders than by direct longitudinal BD studies (51). Overall, these findings support a robust TNF-α–WM association, while the roles of COR and MIF in executive function require validation in larger longitudinal samples.

In this study, patients with BD during manic episodes showed significant improvements in IPS, EF, SAT, and WM at the 8-week follow-up compared with baseline. Although TNF-α, MIF, and COR all showed downward trends, these changes did not reach statistical significance, suggesting that cognitive recovery may not be fully synchronized with changes in peripheral immune-inflammatory and neuroendocrine markers. However, this interpretation should be cautious because nonsignificant biomarker changes may also reflect limited statistical power, short follow-up duration, or delayed biological normalization. Several factors may account for this finding. First, current treatment strategies for BD primarily focus on controlling mood symptoms. The principal mechanisms of commonly used medications, such as second-generation antipsychotics and lithium salts, involve modulation of neurotransmitter systems and related intracellular signaling pathways. Although these agents may exert certain anti-inflammatory effects, sufficient and consistent evidence regarding their direct regulatory effects on chronic inflammatory states and HPA axis dysfunction remains lacking (52, 53). Second, reversal of chronic inflammatory states and restoration of HPA axis homeostasis may require a longer treatment period, and the relatively short follow-up duration in this study may have been insufficient to capture delayed biomarker changes. Finally, the limited sample size may have reduced the statistical power of the study, thereby preventing detection of subtle but clinically meaningful decreases in biomarker levels.

The findings of this study support the inclusion of cognitive impairment as an independent domain in the clinical assessment of BD and further suggest that peripheral biological markers may provide supplementary information for identifying cognitive risk. In future clinical management of patients with BD, incorporating the combined assessment of specific cognitive domains and relevant biomarkers alongside routine symptom evaluations may facilitate the identification of individuals who remain at high risk for cognitive impairment despite improvements in mood symptoms.

Several limitations of this study should be acknowledged. First, the sample size calculation was based on the primary within-group longitudinal analysis of cognitive changes rather than on biomarker–cognition correlation analyses. Therefore, the correlation analyses, particularly the adjusted partial correlations including several covariates, may have been underpowered to detect small-to-moderate associations. Accordingly, the biomarker–cognition findings should be interpreted as exploratory and require validation in larger longitudinal cohorts. Second, the sample size was relatively small and the study was conducted at a single center, which may limit the generalizability of the findings. Third, because of sample size constraints, subgroup analyses according to specific medication types were not performed; therefore, the potential confounding effects arising from differences in the pharmacological mechanisms of various medications could not be excluded. Fourth, the short follow-up period precluded a clear understanding of the long-term trajectories of TNF-α, MIF, and COR in relation to cognitive function and made it difficult to determine the temporal or causal relationships among these variables. Fifth, the underlying mechanisms were inferred solely from the associations between peripheral blood markers and cognitive scores, without the inclusion of neuroimaging measures; therefore, the correspondence between peripheral biomarkers and functional changes in specific brain regions could not be directly validated. Sixth, the 8-week follow-up assessment reflected clinical response rather than full euthymia. Although patients showed a reduction of more than 50% in BRMS scores, a substantial proportion did not reach remission-level manic symptoms according to the operational BRMS criterion. Residual manic symptoms may therefore have influenced cognitive performance and biomarker–cognition associations. Moreover, because euthymia cannot be fully determined by BRMS alone, future studies should define euthymia more rigorously using both manic and depressive symptom measures and should consider conducting sensitivity analyses restricted to patients who achieve full symptomatic remission.

Future research should involve large-sample, multicenter, longitudinal studies with extended follow-up, incorporating neuroimaging, metabolic markers, and more refined repeated-measures designs to further clarify the dynamic changes in TNF-α, MIF, and COR in BD-related cognitive impairment, as well as their potential utility for stratification and prediction.

## Conclusion

This short-term follow-up study showed that patients with BD during acute manic episodes exhibited broad cognitive impairment and elevated plasma TNF-α, MIF, and COR levels compared with healthy controls. After adjustment for covariates and correction for multiple comparisons, TNF-α showed the most robust negative association with working memory at both baseline and follow-up. These findings add longitudinal evidence to the existing literature and suggest that TNF-α-related inflammatory activity may be particularly relevant to working memory impairment in BD, whereas associations involving MIF or cortisol and executive function should be regarded as exploratory.

## Data Availability

The raw data supporting the conclusions of this article will be made available by the authors, without undue reservation.
