# Supplementary material for: Associations of TNF-α, MIF, and cortisol with cognitive function in patients with bipolar disorder during acute manic episodes: a short-term follow-up study
Source: Front Psychiatry. 2026 Jun 29;17:1863310. doi: 10.3389/fpsyt.2026.1863310 (PMC13357933; doi:10.3389/fpsyt.2026.1863310)
Supplement: Supplementary file 1 [file Table1.docx]

**Supplementary Table 1 Correlations of Chlorpromazine-Equivalent Dose With Plasma Biomarkers and Cognitive Performance in the BD Group**

| Variable | *r* | *P* |
| --- | --- | --- |
| MIF | -0.097 | 0.491 |
| TNF-α | -0.005 | 0.972 |
| COR | -0.223 | 0.109 |
| IPS | 0.157 | 0.262 |
| EF | 0.100 | 0.478 |
| SAT | -0.137 | 0.326 |
| WM | -0.075 | 0.593 |

**Abbreviations:** MIF, macrophage migration inhibitory factor; TNF-α, tumor necrosis factor-α; COR, cortisol; IPS, information processing speed; EF, executive function; SAT, sustained attention; WM, working memory.

**Note:** Correlations between chlorpromazine-equivalent dose and plasma biomarkers and cognitive performance were analyzed in the BD group. *P* < 0.05 was considered statistically significant.

**Supplementary Table 2. Comparisons of Plasma TNF-α, MIF, COR, and Cognitive Performance Between the BD and HC Groups at Baseline and Follow-up**

| Variable | Time point | | BD(*n*=53) | HC(*n*=53) | *t* | *P* |
| --- | --- | --- | --- | --- | --- | --- |
| IPS | | Baseline | 44.30±3.95 | 50.02±2.21 | -9.192 | <0.001 |
|  |  | Follow-up | 45.94±4.01 |  | -6.481 | <0.001 |
| EF | | Baseline | 39.64±5.88 | 50.34±5.39 | -9.765 | <0.001 |
|  |  | Follow-up | 42.42±6.48 |  | -6.842 | <0.001 |
| SAT | | Baseline | 45.04±8.50 | 51.79±2.63 | -5.525 | <0.001 |
|  |  | Follow-up | 46.83±7.73 |  | -4.422 | <0.001 |
| WM | | Baseline | 42.66±6.70 | 52.09±5.07 | -8.177 | <0.001 |
|  |  | Follow-up | 45.15±7.01 |  | -5.843 | <0.001 |
| MIF | | Baseline | 35.46±5.26 | 22.96±5.52 | 11.930 | <0.001 |
|  |  | Follow-up | 34.51±4.42 |  | 11.888 | <0.001 |
| TNF-α | | Baseline | 31.86±5.66 | 21.93±5.45 | 9.207 | <0.001 |
|  |  | Follow-up | 30.38±6.00 |  | 7.594 | <0.001 |
| COR | | Baseline | 93.86±14.24 | 54.69±4.01 | 19.275 | <0.001 |
|  |  | Follow-up | 88.39±16.23 |  | 14.679 | <0.001 |

**Abbreviations:** BD, bipolar disorder; HC, healthy controls; IPS, information processing speed; EF, executive function; SAT, sustained attention; WM, working memory; MIF, macrophage migration inhibitory factor; TNF-α, tumor necrosis factor-α; COR, cortisol.

**Note:** Data are presented as mean ± SD. *P* < 0.05 was considered statistically significant.

**Supplementary Table 3. ANCOVA of baseline cognitive performance in the BD and HC groups after adjustment for demographic covariates**

| Cognitive domain | Levene’s test, *P* | Group effect, F(1,100) | *P* value | Partial η² |
| --- | --- | --- | --- | --- |
| IPS | <0.001 | 43.769 | <0.001 | 0.304 |
| EF | 0.193 | 51.504 | <0.001 | 0.340 |
| SAT | <0.001 | 6.507 | 0.012 | 0.061 |
| WM | 0.396 | 30.770 | <0.001 | 0.235 |

**Abbreviations:** BD, bipolar disorder; HC, healthy controls; IPS, information processing speed; EF, executive function; SAT, sustained attention; WM, working memory.

**Notes:** Analysis of covariance (ANCOVA) was performed with group and sex as fixed factors and age, body mass index (BMI), and years of education as covariates. The F and P values shown refer to the main effect of group after adjustment for the above covariates. A two-sided *p* value < 0.05 was considered statistically significant. Levene’s test suggested violation of homogeneity of variance for IPS and SAT; therefore, these adjusted results should be interpreted with caution.

**Supplementary Table 4. Correlations of Cognitive Performance with Plasma Biomarkers in the BD Group**

| Cognitive domain | MIF | | TNF-α | | COR | |
| --- | --- | --- | --- | --- | --- | --- |
|  | *r* | *P value* | *r* | *P value* | *r* | *P value* |
| Baseline |  |  |  |  |  |  |
| IPS | 0.031 | 0.825 | 0.073 | 0.604 | -0.053 | 0.709 |
| EF | -0.084 | 0.548 | 0.091 | 0.515 | -.411 | 0.002 |
| SAT | 0.033 | 0.816 | 0.246 | 0.076 | -0.025 | 0.858 |
| WM | -0.008 | 0.957 | -.467 | <0.001 | -0.189 | 0.174 |
| Follow-up |  |  |  |  |  |  |
| IPS | -0.006 | 0.966 | 0.099 | 0.483 | 0.187 | 0.180 |
| EF | -.429 | 0.001 | -.321 | 0.019 | -.441 | 0.001 |
| SAT | -0.016 | 0.908 | 0.147 | 0.294 | 0.119 | 0.395 |
| WM | -0.222 | 0.110 | -.463 | <0.001 | -0.155 | 0.268 |

**Abbreviations:** IPS, information processing speed; EF, executive function; SAT, sustained attention; WM, working memory; MIF, macrophage migration inhibitory factor; TNF-α, tumor necrosis factor-α; COR, cortisol.

**Note:** *P* < 0.05 was considered statistically significant.
